# P-1842. Clinical Characteristics of Rat Hepatitis E Virus (Rocahepevirus ratti) Infection: an emerging agent of viral hepatitis in humans

**DOI:** 10.1093/ofid/ofaf695.2011

**Published:** 2026-01-11

**Authors:** Siddharth Sridhar, Shusheng Wu, Cyril Yip, Estie Hon-Kiu Shun, Tsz Chung Wong, Pak Yui Ng, Zhiyu Li, Jianwen Situ, Stanley Ho

**Affiliations:** The University of Hong Kong, Hong Kong, Hong Kong; University of Hong Kong, Hong Kong, Not Applicable, Hong Kong; The University of Hong Kong, Hong Kong, Hong Kong; University of Hong Kong, Hong Kong, Not Applicable, Hong Kong; University of Hong Kong, Hong Kong, Not Applicable, Hong Kong; University of Hong Kong, Hong Kong, Not Applicable, Hong Kong; University of Hong Kong, Hong Kong, Not Applicable, Hong Kong; University of Hong Kong, Hong Kong, Not Applicable, Hong Kong; University of Hong Kong, Hong Kong, Not Applicable, Hong Kong

## Abstract

**Background:**

*Rocahepevirus ratti* genotype 1 (rat hepatitis E virus; rHEV) is a rodent virus that is highly divergent to conventional human-infecting hepatitis E virus (*Paslahepevirus balayani*; bHEV). In 2018, we reported the first case of human rHEV infection. Since then, cases have been reported globally. However, the characteristics of human rHEV infection are poorly understood. We conducted a clinical and epidemiological assessment of this emerging zoonosis in Hong Kong.

**Table 1:**
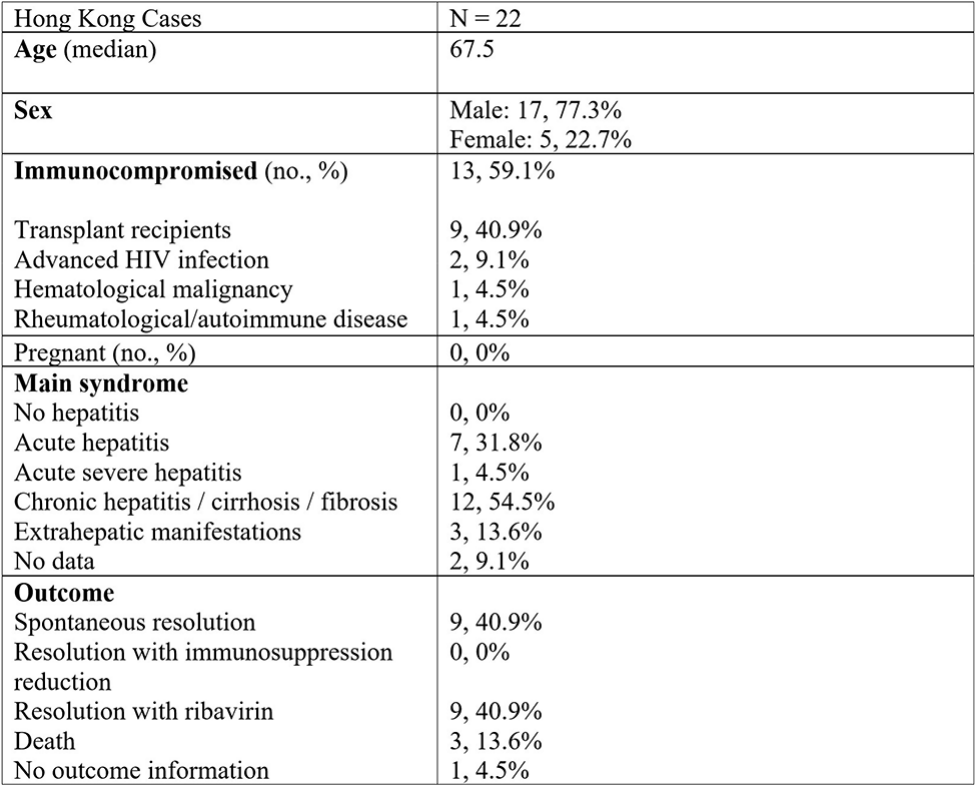
Clinical characteristics of Rocahepevirus ratti cases identified in Hong Kong

**Figure 1:**
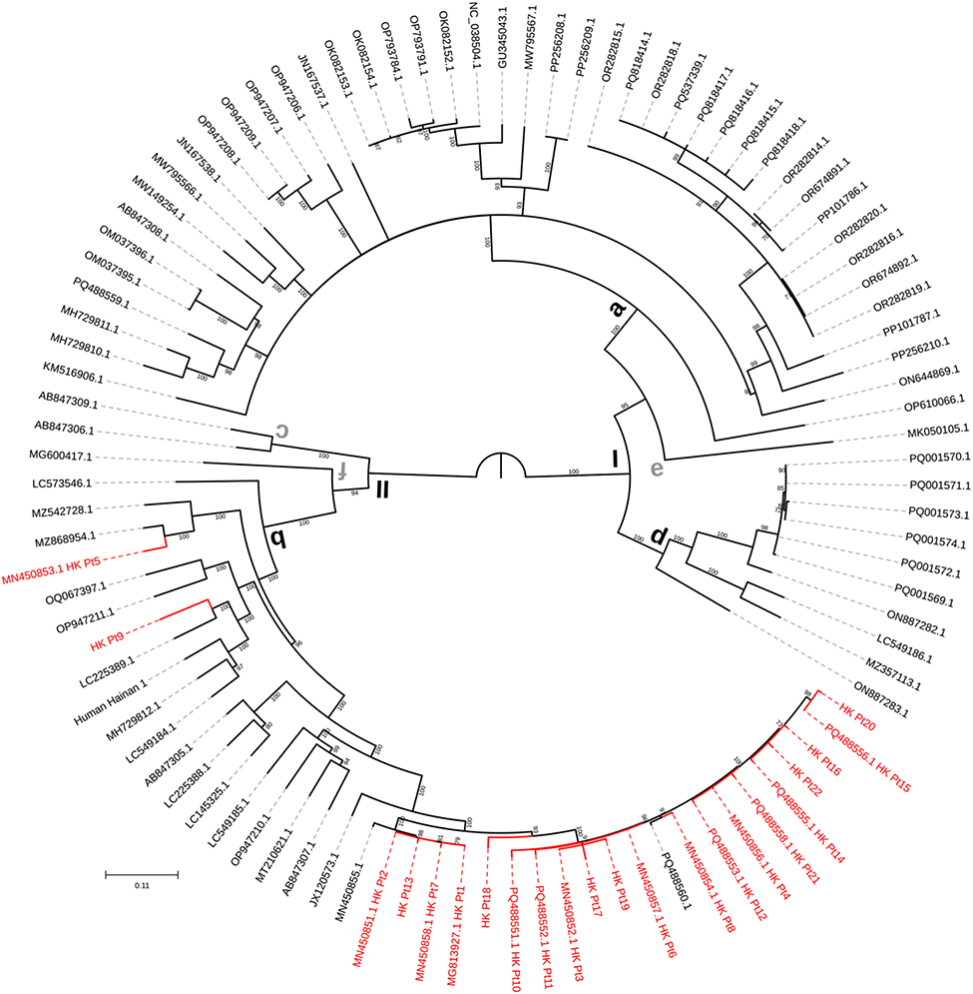
Rocahepevirus ratti phylogeny inferred using the Maximum Likelihood Estimation method with GTR+F+R4 model on nucleotide sequences. Hong Kong strains are highlighted in red. Ultrafast bootstrap supports are labelled on each branch and branches with less than 70% support are polytomised.

**Methods:**

This study was conducted from 2018 to 2025 at a reference laboratory offering rHEV testing in Hong Kong. rHEV RNA was detected with a validated qRT-PCR assay. Positive samples were sequenced using Sanger and NGS approaches. Population bHEV and rHEV IgG seroprevalence was measured on a biobank of plasma samples using a validated enzymatic immunoassay (EIA) protocol.

**Figure 2:**
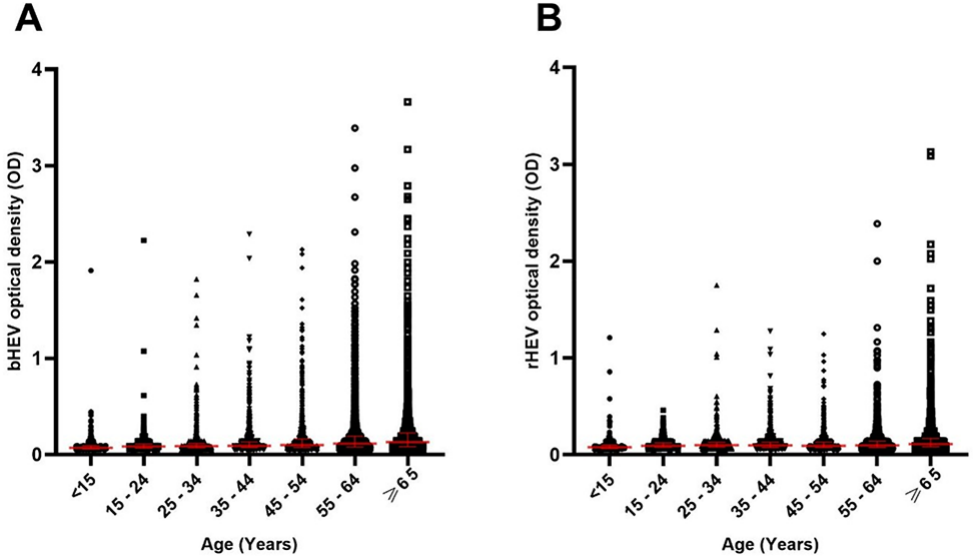
Species-specific hepatitis E virus IgG enzymatic immunoassay results on a large plasma biobank. Trend of Paslahepevirus balayani (A) and Rocahepevirus ratti (B) IgG enzymatic immunoassay optical densities with increasing age. Bars represent median and interquartile ranges.

**Results:**

Over a 7-year period, we received 2,018 deduplicated requests from patients with hepatitis for HEV qRT-PCR. Of these, 75 (3.7%) tested positive for bHEV and 16 (0.8%) tested positive for rHEV RNA. An additional six rHEV cases were identified by surveillance in transplant recipients and other laboratories in Hong Kong. Clinical characteristics of these 22 patients are presented in table 1. Compared to bHEV, rHEV caused milder hepatitis, but affected immunocompromised persons more commonly with high rates of chronicity. Nine chronic rHEV patients received ribavirin with sustained virological response. All cases belonged to a single subtype and 20/22 were monophyletic with Hong Kong rat-derived rHEV strains (figure 1). No patient had directly contacted rodents, but three saw rats near their residence or workplace. A biobank of 10,691 plasma samples were tested in rHEV and bHEV IgG EIAs. The bHEV seroprevalence was 463/10,691 (4.3%) and that of rHEV was 54/10,691 (0.5%). Median ODs in both bHEV and rHEV EIAs rose with age (figure 2); age was associated with rHEV seropositivity (p < 0.001). Unlike bHEV, sex, residential district, intravenous drug use, and hepatitis C infection were not associated with rHEV IgG seropositivity.

**Conclusion:**

We present evidence for continuous low-level exposure of humans to rHEV. Immunocompromised hosts are at risk of chronic rHEV infection. Ribavirin is an effective treatment for this condition.

**Disclosures:**

All Authors: No reported disclosures

